# Is plasma calcium concentration implicated in the development of critical illness polyneuropathy and myopathy?

**DOI:** 10.1186/cc10505

**Published:** 2011-10-21

**Authors:** Dimitri Anastasopoulos, Antonios Kefaliakos, Argyris Michalopoulos

**Affiliations:** 1 Department of Physiology and Clinical Neurophysiology, School of Nursing, University of Athens, 8 Tetrapoleos Street, 11527 Goudi, Athens, Greece; 2 Department of Neurology, Henry Dunant Hospital, 107 Mesogeion Avenue, 11526 Athens, Greece; 3 Intensive Care Unit, Henry Dunant Hospital, 107 Mesogeion Avenue, 11526 Athens, Greece

## Abstract

**Introduction:**

This prospective study investigated whether plasma ionized calcium concentration abnormalities and other electrolyte disturbances represent risk factors for the development of critical illness polyneuromyopathy (CIPNM) in ICU patients.

**Methods:**

One hundred and ninety consecutive adult critically ill patients with prolonged ICU stay (longer than 7 days) were prospectively evaluated. Patients with acute weakness and/or weaning difficulties were subjected to extensive electrophysiological measurements in order to establish the diagnosis of CIPNM. All recognized and/or possible risk factors for development of CIPNM were recorded.

**Results:**

The diagnosis of CIPNM was confirmed in 40 patients (21.05%). By applying a logistic regression model, hypocalcemia (*P *= 0.02), hypercalcemia (P = 0.01) and septic shock (*P *= 0.04) were independently associated with the development of CIPNM in critically ill patients.

**Conclusions:**

We found that septic shock and abnormal fluctuations of plasma Ca^2+ ^concentration represent significant risk factors for the development of CIPNM in critically ill patients.

## Introduction

Acutely acquired neuromuscular dysfunction is a common phenomenon among critically ill patients in the ICU, with a prevalence ranging between 25 and 60% depending upon the criteria used for the diagnosis, the population studied and the timing of examination [1]. Critical illness polyneuropathy and critical illness myopathy have been established as separate entities of muscular weakness leading to considerable weaning difficulties and prolonged ICU stay. These two entities often coexist [2] and have been related to systemic inflammatory response syndrome and sepsis [3]. Several risk factors, such as the administration of neuromuscular blocking agents, aminoglycosides and high-dose corticosteroids, have so far frequently but controversially been considered [4-7].

In recent years there has been considerable interest in the role of intracellular calcium homeostasis disturbances as an early event leading to cell injury or death. Calcium is an important intracellular messenger and regulator of cell function. Calcium is essential for the excitation-contraction coupling in muscle, neurotransmission, digestive enzyme activation, inhibition of ATP synthesis and free radical production. Intracellular Ca^2+ ^homeostasis of skeletal muscle fibers in mice is altered during sepsis and this has been implicated in the pathogenesis of critical illness polyneuropathy/myopathy (CIPNM) [8].

Very little is known regarding the association of extracellular calcium concentration abnormalities with the development of neuromuscular dysfunction in critically ill patients. This lack of information is surprising, as ionized hypocalcemia or hypercalcemia are not uncommon in patients treated in ICUs and have been shown to correlate with increased mortality [9]. It has long been established that patients with chronic hypocalcemia due to parathyroid gland hypofunction may develop myopathy, and moreover the degree of change in muscles has been found to be related to the severity of hypocalcemia [10,11]. The exact pathophysiological mechanism of this relationship, however, has not yet been elucidated. There is also experimental evidence from an animal model that a reduced Ca^2+ ^plasma concentration elevates creatine kinase due to skeletal muscle injury [12]. Also, chronic hypercalcemia that develops secondary to hyperparathyroidism produces many psychiatric and cognitive symptoms, as well as a reversible myopathy [13].

The present study investigated several possible risk factors associated with the development of CIPNM in critically ill patients. Special emphasis was given to patients' plasma ionized calcium concentration fluctuations and other serum electrolyte abnormalities since they alter membrane excitability.

## Materials and methods

One hundred and ninety consecutive patients were prospectively enrolled in the study protocol. The patients were evaluated on a daily basis during a 9-month period (from January 2008 to October 2008) in the 24-bed multidisciplinary ICU of Henry Dunant Hospital, Athens, Greece.

The study protocol was approved by the Ethics and Research Committee of Henry Dunant Hospital. All of the procedures used were in accordance with the recommendations found in the Helsinki Declaration of 1975. There was no change made during the study period to personnel, surgical procedure, protocol for general anesthesia or management of patients in the ICU. For this reason, a consent form was not obtained.

Included as participants were adult patients (age > 18 years old) who remained hospitalized in the ICU for more than 7 days, independent of cause. Excluded were patients with pre-existing neuromuscular and renal disease as well as those with adrenal insufficiency and calcium metabolism dysfunction.

### Study protocol

The data collected prospectively for each patient included demographics, medical history, and several variables thought to be associated with the pathogenesis of CIPNM - such as Acute Physiology and Chronic Health Evaluation II score recorded on the eighth ICU day, Sequential Organ Failure Assessment score recorded on the first day of documentation of nosocomial infection and/or sepsis syndrome, development of sepsis syndrome, and administration of aminoglycosides, corticosteroids, inotropes or neuromuscular blocking agents for more than 1 day. Serum ionized Ca^2+ ^concentrations, serum Na^+ ^and K^+ ^concentrations, serum glucose levels and arterial blood pH included in the routine laboratory set were recorded every 3 hours from the first ICU day. These variables were analyzed by means of an ABL 800 FLEX Blood Gas Analyzer (Radiometer, Copenhagen, Denmark). Serum ionized Ca^2+ ^concentrations and pH values were corrected for pH 7.40. In addition, we recorded all complications that occurred during the ICU stay, the length of the ICU stay, the patient's outcome (death or discharge), and the cause of any death. The above variables were evaluated daily until either CIPNM was diagnosed or the patient was discharged from the ICU.

The tested risk factors for CIPNM were chosen *a priori *by investigator consensus and based on published articles until December 2007.

Every patient was evaluated once daily for muscle weakness. In cooperative patients, 12 muscle groups were assessed: wrist flexion, forearm flexion, shoulder abduction, knee extension, hip flexion and foot extension. Patients with brain injury or these too heavily sedated to understand simple orders were tested by evaluating the muscle tone. When weaning difficulties appeared (that is, when patients were unable to be definitely released from mechanical ventilation) and/or the flaccid weakness was bilateral, involving upper and lower extremities (resulting in more than 20% reduction of the Medical Research Council score in cooperating patients) [14], extensive electrophysiological investigations were undertaken. All patients showing clinical features of CIPNM (for example, weaning difficulty or flaccid weakness in upper and low extremities) were assessed by electromyography screening.

Electrolyte disturbances were corrected by the intensivists, independently of the study protocol. Cut-off values were considered for ionized Ca^2+ ^< 0.9 mmol/l, Na^+ ^< 130 mmol/l, and K^+ ^< 3.4 mmol/l. The correction of electrolyte disturbances was made by senior intensivists, who were blinded to study parameters, with intravenous administration of electrolyte solutions such as CaCl_2 _(10 ml of 10%) and KCl (10 ml of 10%).

### Definitions

The diagnosis of CIPNM was confirmed by markedly reduced and often prolonged compound muscle action potentials (peroneal and ulnar nerves bilaterally) and abundant, widespread spontaneous activity with fibrillation potentials and positive sharp waves on conventional needle electromyography [15]. Motor unit potentials were in most cases polyphasic, of low amplitude and short duration (myopathic). Both peripheral and proximal muscles of the upper and lower extremities were investigated (tibialis anterior, vastus lateralis, deltoideus, and interosseus dorsalis I). No attempt was made to differentiate between neuropathy and myopathy by directly stimulating the muscles. Entrapment syndromes, especially at the elbow, did not represent a matter of concern in establishing the diagnosis because conduction velocity slowing and electromyography findings were expected to be locally isolated.

Electrolyte disturbances were thought significant only if they had lasted at least 36 hours. Normal values were considered 1.16 to 1.30 mmol/l for ionized Ca^2+^, 134 to 149 mmol/l for ionized Na^+^, and 3.5 to 5.4 mmol/l for ionized K^+ ^[16]. Acute hyperglycemia was defined as serum glucose levels above 180 mg/dl [17]. Sepsis syndrome and septic shock were defined based on standard criteria [18].

### Statistical analysis

The data were analyzed using SPSS 17 for Windows (SPSS Inc., Chicago, IL, USA). The differences were calculated using Fisher's exact test for dichotomous or categorical variables and the two-sample Wilcoxon test for continuous variables. A backward stepwise regression analysis was used to identify the set of independent variables that contributed significantly to the fit of the model (removing criterion, *P *> 0.20). When a multilevel categorical variable was found to be significant, tests were conducted to determine whether some levels could be combined. The dependent variable was binary, with one denoting CIPNM and zero denoting no development of CIPNM. Values are reported as number (percentage), mean ± standard deviation, or median (interquartile range) as appropriate. Significance was set at *P *< 0.05. All reported *P *values are two-sided.

## Results

Out of 968 patients admitted to the ICU during the study period (690 males, 278 females, mean age 65.5 ± 14.8), 40 patients (4.1%) developed CIPNM. In the subgroup of critically ill patients with ICU length of stay longer than 7 days (*n *= 190, 66.8% males) and mean age 66.9 ± 15.4 years, 40 patients (21%) developed CIPNM. The main causes of ICU admission for patients with prolonged ICU length of stay (*n *= 190) are presented in Table 1. The mean Acute Physiology and Chronic Health Evaluation II and Sequential Organ Failure Assessment scores for all patients (*n *= 190) were 21.7 ± 7.0 and 7.9 ± 4.1, respectively. Out of 190 patients enrolled in the study protocol, 142 patients (74.7%) improved and were discharged from the ICU. The median ICU length of stay of survivors (*n *= 142) was 16 days. No patient in the subgroup of patients with length of ICU stay shorter than 7 days developed CIPNM.

**Table 1:** Causes of ICU admission for 190 patients

Cause	Number of patients
Heart failure	28
Chronic obstructive pulmonary disease	25
Infection	39
Cerebrovascular event	15
Trauma	8
Postoperative support	55
Other	20

In the group of patients who developed a documented CIPNM during their ICU stay, the length of mechanical ventilation was 32.88 ± 22.2 days - compared with 14.4 ± 9.0 days for patients with prolonged ICU stay (*n *= 150) who did not develop CIPNM (*P *< 0.05). Thirteen out of 40 patients (32.5%) with CIPNM died in the ICU. The mean length of ICU stay of survivors (*n *= 27) was 30.7 ± 18.5 days.

**Figure 1:**
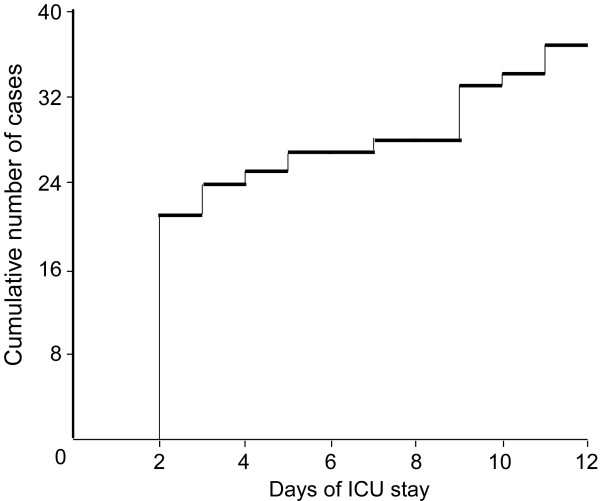
**Emergence of the first episode of prolonged hypocalcemia**. Time point of emergence of the first episode of prolonged hypocalcemia in 37 patients who developed critical illness polyneuromyopathy.

During the episodes of prolonged hypocalcemia or hypercalcemia, the ionized Ca^2+ ^serum concentration varied between 0.33 and 1.14 mmol/l and between 1.35 and 1.53 mmol/l, respectively. Severe hypocalcemia (ionized Ca^2+ ^serum concentration < 0.90 mmol/l) was found in 13 patients. The first episode of hypocalcemia occurred within the first few days in the ICU (median ICU length of stay 2 days, upper quartile 7 days; Figure 1). All of these episodes occurred a few days prior the development of CIPNM. The time points of hypocalcemia and CIPNM diagnosis did not, however, correlate significantly.

Episodes of prolonged hypocalcemia were recorded in 101 out of 126 patients with septic shock. Of these patients (*n *= 126), 33 patients (26.19%) developed CIPNM.

Univariate statistical analysis showed that among the 15 variables examined as possible risk factors for development of CIPNM in critically ill patients five variables were significant. For these risk factors, logistic regression analysis showed that hypocalcemia, hypercalcemia and septic shock were major determinants significantly and independently associated with the development of CIPNM in adult patients in the ICU (Table 2).

**Table 2:** Risk factors for development of critical illness polyneuromyopathy

Variable	Patients with CIPNM (*n *= 40)	Patients without CIPNM (*n *= 150)	*P *value
Age	68.7 ± 15.7	66.4 ± 15.9	0.55
Female gender	24	39	0.002
APACHE II score	23.7 ± 6.5	20.1 ± 7	0.04
SOFA score	8.4 ± 4.1	7.8 ± 4.2	0.16
Aminoglucosides	20	52	0.18
Corticosteroids	28	102	0.84
NMBA	16	40	0.35
Septic shock	33	93	0.04
Hyperglycemia	10	43	0.66
Hyperkalemia	0	1	1
Hypokalemia	5	14	0.49
Hypernatremia	6	9	0.14
Hyponatremia	7	30	0.89
Hypercalcemia	4	3	0.01
Hypocalcemia	37	114	0.02

APACHE, Acute Physiology and Chronic Health Evaluation; CIPNM, critical illness polyneuromyopathy; NMBA, neuromuscular blocking agents; SOFA, Sequential Organ Failure Assessment.

**Figure 2:**
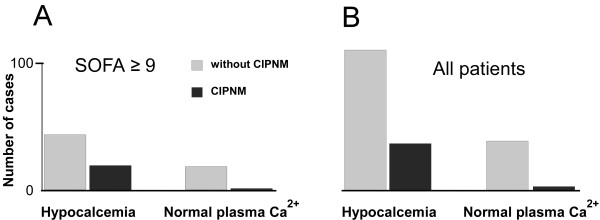
**Episodes of prolonged hypocalcemia or hypercalcemia in ICU patients**. **A **Patients with more severe critical illness (Sequential Organ Failure Assessment (SOFA) ≥9, *n *= 76) but normal plasma Ca^2+ ^concentration did not develop critical illness polyneuromyopathy (CIPNM). In contrast, CIPNM was frequently observed in patients of the same group presenting with hypocalcemia. **B **Hypocalcemia/CIPNM cases in all 190 patients.

The relationship between hypocalcemia and CIPNM was further explored by identifying a group of patients containing those most severely affected (Sequential Organ Failure Assessment score ≥9, *n *= 76). Hypocalcemia was found to be significantly related to the development of CIPNM (*P *= 0.03). Thus, only one patient with more severe critical illness but normal plasma calcium levels developed CIPNM (Figure 2A). In contrast, approximately one-third of hypocalcemic patients belonging to this group of severely affected patients developed CIPNM.

**Figure 3:**
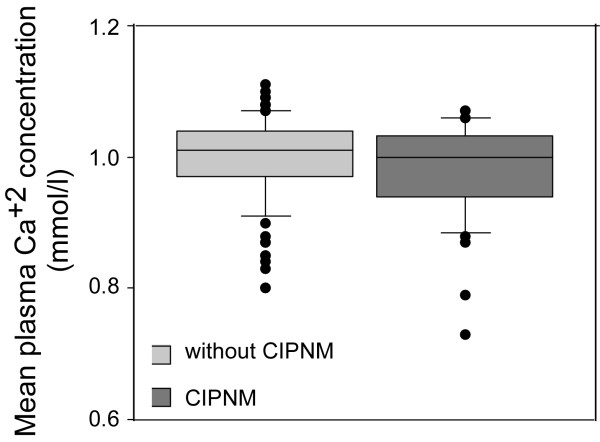
**Plasma calcium concentrations during prolonged hypocalcemia**. Mean Ca^2+ ^concentration during the episode of prolonged hypocalcemia in patients with and without critical illness polyneuromyopathy (CIPNM).

The severity of hypocalcemia, however, did not correlate with the severity of CIPNM. Noticeably, seven out of the 13 patients with severe hypocalcemia (lower than 0.90 mmol/l) did not develop CIPNM and the mean Ca^2+ ^concentration during the episode of hypocalcemia in patients with CIPNM was not significantly lower than in those without CIPNM (Figure 3).

The diagnosis of CIPNM was confirmed several days (mean ICU length of stay 15.8 ± 6.0) after ICU admission.

## Discussion

In this original study, we found that septic shock and hypocalcemia represent significant risk factors for the development of CIPNM in adult critically ill patients. Ionized hypocalcemia has been shown to represent a common laboratory abnormality among critically ill patients and to be associated with increased mortality [8,19]. Our prospective study, based on a large unselected patient population of a mixed medical-surgical ICU, demonstrated that hypocalcemia represents a significant predisposing factor for the development of CIPNM.

On the other hand, both ionized hypocalcemia and CIPNM may be related to sepsis syndrome. One might therefore argue that the abnormal fluctuations of plasma Ca^2+ ^concentration are not the cause but simply a consequence of severe sepsis not directly related to CIPNM. Alternative explanations cannot be excluded, however, as their relationship was confirmed equally in a subgroup of more severely affected patients.

One of the proposed mechanisms for hypocalcemia is influx and intracellular accumulation of calcium ions [20]. Increased concentrations of Ca^2+ ^into muscle cells may represent the critical link in the pathophysiology of CIPNM; proteolysis of myofibrillar structure proteins, mainly myosin thick filaments, is dependent on Ca^2+ ^release from intracellular stores as the administration of dantrolene is able to block this response to sepsis in rat skeletal muscle [21].

Muscle membrane hypoexcitability has been recently associated with sepsis, and may be implicated with the development of CIPNM [22]. Hypocalcemia increases neuronal membrane excitability by reducing the threshold of voltage-dependent Na^+ ^channel activation [23] and by directly interacting with channel gating machinery [24]. In CIPNM muscle membrane, however, excitability is reduced. Noticeably, the molecular mechanisms by which Ca^2+ ^may affect muscle membrane excitability and the signals it may convey to the cell interior (that is, by means of extracellular calcium-sensing receptors) are, in contrast to the extensively studied intracellular actions, less well investigated.

Apart from persistent hypocalcemia, we found that septic shock was strongly correlated with the development of CIPNM in ICU patients. This finding is in accordance with previous original studies [25-27]. Among several other risk factors implicated, diabetes mellitus seems to play a crucial role in critical illness polyneuropathy/critical illness myopathy. Bird reports that intensive insulin therapy in diabetic patients in the ICU appears to reduce the likelihood of developing CIPNM [28]. On the other hand, Rich and colleagues showed that high-dose corticosteroids and a number of factors seem to modify membrane excitability [29,30]. This effect has been attributed to a shift in the voltage dependence of sodium channel fast inactivation towards more negative potentials in conjunction with depolarization of the resting membrane potential. Reduced sodium channel availability has also been shown by investigating muscle membrane excitability in CIPNM with velocity recovery cycles [31]. One should note, however, that persistent serum hypocalcemia acts in the neuronal membrane by means of an unknown pathophysiological mechanism.

Not only hypocalcemia but also prolonged hypercalcemia were found to be significant predisposing factors in our study. This observation may be not completely surprising, as chronic elevation of Ca^2+ ^plasma concentration in primary hyperparathyroidism may result in muscle weakness [32]. Hypercalcemia may also result as a consequence of malignancy, infections or immobilization. In a consecutive series of 100 cases with primary hyperparathyroidism, patients with neuromuscular symptoms had significantly higher plasma calcium levels than the rest of the group [33]. The mechanism and pathology of these symptoms have not yet been elucidated. Consequently, since prolonged hypercalcemia was relatively infrequent in our patient group, we feel that its exact significance should be examined in larger patient populations.

## Conclusions

We found that septic shock and hypocalcemia represent independent risk factors for the development of CIPNM in critically ill patients. Future research may be warranted to confirm these findings in larger patient populations and to investigate the molecular mechanism linking extracellular with intracellular calcium homeostasis abnormalities.

## Key messages

• Long-lasting decreased levels of plasma Ca^2+ ^concentration are significantly correlated with CIPNM in adult patients in the general ICU.

• Apart from hypocalcemia, septic shock represents another significant risk factor for ICU-acquired CIPNM.

## Abbreviations

CIPNM: critical illness polyneuromyopathy.

## Competing interests

The authors declare that they have no competing interests.

## Authors' contributions

DA conceived and organized the project, executed neurological and electrophysiological investigations and wrote the final manuscript. AK collected the clinical and biochemical data, performed statistical analysis and wrote the first draft. AM participated in the design of the study and critically reviewed the manuscript. All authors read and approved the final manuscript.
